# Evaluating LLMs’ grammatical error correction performance in learner Chinese

**DOI:** 10.1371/journal.pone.0312881

**Published:** 2024-10-30

**Authors:** Sha Lin

**Affiliations:** Yantai Institute of Technology, Yantai, China; University of the Chinese Academy of Sciences, CHINA

## Abstract

Large language models (LLMs) have recently exhibited significant capabilities in various English NLP tasks. However, their performance in Chinese grammatical error correction (CGEC) remains unexplored. This study evaluates the abilities of state-of-the-art LLMs in correcting learner Chinese errors from a corpus linguistic perspective. The performance of LLMs is assessed using standard evaluation metrics of MaxMatch score. Keyword and key n-gram analyses are conducted to quantitatively explore linguistic features that differentiate LLM outputs from those of human annotators. LLMs’ performance in syntactic and semantic dimensions is further qualitatively analyzed based on these probes of keywords and key n-grams. Results show that LLMs achieve a relatively higher performance in test datasets with multiple annotators and low performance in those with a single annotator. LLMs tend to overcorrect wrong sentences, under the explicit prompt of the “minimal edit” strategy, by using more linguistic devices to generate fluent and grammatical sentences. Furthermore, they struggle with under-correction and hallucination in reasoning-dependent situations. These findings highlight the strengths and limitations of LLMs in CGEC, suggesting that future efforts should focus on refining overcorrection tendencies and improving the handling of complex semantic contexts.

## Introduction

A critical aspect of improving learners’ proficiency in Chinese as a foreign language is grammatical error correction (GEC), a specialized area within the domain of both natural language processing and applied linguistics involving the automatic detection and correction of grammatical errors in learners’ written texts. With the current global rise in the number of students learning Chinese, the demand for efficient, automated systems capable of accurately identifying and correcting Chinese grammatical errors is increasing.

Current research in GEC has predominantly featured English, experiencing significant advancements with NLP technologies like sequence-to-sequence (seq2seq) [1–3] and sequence-to-edit (seq2edit) [4–6] achieving state-of-the-art results in major benchmarks. However, these methods often require extensive labeled datasets, posing difficulties in scenarios where such resources are scarce. Despite considerable advancements, applying these systems to Chinese presents notable challenges including the scarcity of high-quality parallel data and the intrinsic complexities of the Chinese language [7]. While Chinese GEC (CGEC) has been somewhat explored [8], accurately resolving the learner Chinese errors remains a daunting task, underscoring a demand for more refined correction systems. LLMs have shown impressive capabilities in various natural language processing tasks due to their ability to generalize across languages and tasks through in-context learning, thus addressing low-resource challenges effectively. Given the proficiency of LLMs in overcoming these data limitations, the increasing exploration of LLMs in CGEC showed that overcorrection is a major problem [9]. LLMs present a promising avenue for enhancing GEC systems, as recent studies have begun leveraging these models to improve the accuracy of corrections. However, we are still knowing little about how errors are over or under-corrected by LLMs and how well they could do in the different CGEC tasks. To the best of our knowledge, several researchers conducted an evaluation of ChatGPT’s performance in the English GEC [10–15], and no studies have been done on LLMs’ CGEC abilities.

When it comes to the ability of LLMs in CGEC, a noticeable gap persists; much like earlier research, current approaches often overlook the necessity of analyzing and evaluating the linguistic qualities of model outputs. Current efforts typically focus on refining algorithms without a deep understanding of the lexical, syntactical, and semantic nuances being processed. This oversight restricts the potential feedback loop where insights from linguistic analysis could inform and inspire further model improvements. This study evaluates three mainstream LLMs’ performance in CGEC from a corpus linguistics perspective to have a comprehensive understanding of LLMs’ performance in CGEC, extend the scope of CGEC research by focusing on qualitative and quantitative analysis of the generated texts in CGEC, and enrich the analytical methods such as keywords and key n-gram analysis in corpus linguistics. The main contributions of this study are twofold. Firstly, it is the first study to evaluate mainstream LLMs for CGEC. Secondly, it is the first to combine detailed corpus linguistics analysis and qualitative methods to examine how these models perform in terms of lexical, syntax, and semantic aspects.

In the next section, a brief literature review will be carried out to identify existing gaps in CGEC for learners, particularly emphasizing the limited exploration of large language models in this domain. Following this, in the “Methods” section, the datasets, evaluation metrics, and corpus linguistic analysis of the LLMs outputs will be presented. The study employs the APIs of leading LLMs—namely ChatGPT, Ernie-4 (Wenxinyiyan from Baidu), and Llama3—to correct grammatical errors and systematically evaluate their performance. Subsequently, the next section will present the results of keywords and key n-grams analysis to assess the models’ effectiveness across lexical, syntactic, and semantic dimensions. Finally, the study will qualitatively analyze the findings in depth, highlighting the strengths and limitations of LLMs in CGEC, and proposing implications and recommendations for future research and advancements in the task of correcting Chinese grammatical errors.

## Literature review

### LLMs in English GEC

GEC has developed with advanced natural language processing technologies from rule-based, classifier, and statistical machine translation to deep learning models, particularly those based on the transformer architecture. By treating GEC as a machine translation or sequence-to-sequence task, pretrained transformer models map erroneous text to corrected forms using extensive authentic data and complex architectures. Studies [16, 17] have demonstrated state-of-the-art results, highlighting these models’ effectiveness in addressing diverse grammatical errors. Alternatively, studies using the sequence-to-edit approach [6, 7, 18], which converts input sentences into a series of edit operations, have gained popularity and enhanced correction accuracy by focusing directly on errors and their corrections, resulting in faster and more precise outputs.

The emergence of Generative Pre-trained Transformer (GPT) models has led to remarkable progress in the field of large language models (LLMs). ChatGPT can outshine existing models in grammatical error correction for English [10], Chinese [19], Japanese [20], Korean [21], and Arabic [22], implying its potential as a valuable tool for second language learners striving to enhance their writing accuracy and fluency. A current trend in English GEC involves writing prompts for LLMs such as GPT3, GPT-3.5, or GPT-4 that would generate grammatical corrections [10, 23, 24]. Coyne et al. [23] assessed the GEC capabilities of GPT-3.5 (text-DaVinci-003) and GPT-4 (GPT-4-0314) using major benchmarks like BEA-2019 and JFLEG. It explores both zero-shot and few-shot prompt settings and highlights that GPT-4 achieves a new high score on the JFLEG benchmark. Through detailed analysis, the study reveals that GPT models tend to make fluency edits and over-corrections. Human evaluations indicate that GPT-4 produces fewer under-corrections but more over-corrections compared to traditional models, showcasing its ability to generate grammatically correct and fluent text. The use of LLMs like GPT-3.5 and GPT-4 has shown promising results, yet the field still remains relatively underexplored. Key issues like prompt engineering, model consistency, and thorough performance assessments are major challenges. Particularly, consistency across different LLMs and their effectiveness in correcting grammatical, semantic, and even pragmatic errors require further examination. Further research is needed to explore more models like LLaMA3, addressing these challenges and expanding the application to ensure robust and reliable performance.

### LLMs in Chinese GEC

In the NLPCC-2018 shared task [2], many systems adopt Seq2Seq models based on RNN/CNN [25, 26]. Recent work adopts diverse approaches of Transformer [27, 28], Seq2Edit [29, 30], and LLMs [19, 29] to achieve state-of-the-art performance. Of the latest LLMs-based studies, Fan et al. [19] introduce GrammarGPT to CGEC using a hybrid dataset of ChatGPT-generated and manually annotated data. By leveraging instruction tuning and innovative data augmentation techniques, GrammarGPT demonstrates the potential of open-source language models in specialized linguistic tasks. For learner Chinese GEC, MuCGEC [30] introduces a comprehensive dataset designed to improve the evaluation of CGEC models. Li et al. [31] explore the application Baichuan-13B-Chat, using instruction tuning to refine their performance on CGEC tasks. The study finds that incorporating universal instructions and leveraging both full-parameter fine-tuning and LoRA (a parameter-efficient fine-tuning technique) can enhance the models’ correction capabilities, underscoring the importance of tailored instruction data. Besides modeling optimization, techniques like data augmentation [27, 28] and model ensemble [29] have proven to be very useful for CGEC.

Recent advancements in CGEC have demonstrated significant and dynamic progress, driven by innovative model architectures, fine-tuning strategies, and comprehensive datasets. This brief overview highlights the current dominance of LLM-based systems. Despite their effectiveness, these systems often depend on substantial computing resources and human-annotated training data. These systems generally treat CGEC as a translation or text generation task, neglecting the specific aspects of grammatical error correction as highlighted by Bryant et al. [32]. With the increasing reliance on neural language models, researchers risk becoming dependent on these less transparent “black box” systems, potentially losing control over the specific CGEC tasks. There is a notable lack of focus on the inefficiencies and limitations of these systems, as well as an insufficient understanding of the real performance of other influential LLMs, such as GPT4, LLaMA3, and ERNIE4. To effectively manage CGEC and address the needs of Chinese foreign language learners through a more transparent approach, it is essential for researchers to assess these models from linguistic and language learning perspectives. Researchers must explore the operational mechanisms specific to CGEC and devise strategies to improve their effectiveness.

### Error analysis in Chinese GEC

Error analysis in GEC is critical for understanding and improving model performance. Fang et al. [10] evaluated ChatGPT on the CoNLL14 test set, focusing on fluency, minimal edits, over-correction, and under-correction. They found that ChatGPT exhibited superior fluency, correcting sentences freely and diversely rather than using minimal edits. While it had fewer under-corrections, it was prone to over-corrections. Similarly, Wu et al. [14] conducted a human evaluation, revealing that ChatGPT produced the most over-corrections but had the fewest under-corrections and fewer mis-corrections compared to other systems.

In the context of CGEC, error analysis is equally vital. Li et al. [26] examined a CNN model, identifying four main error types: word-level errors in common words, contextual errors, challenging verb corrections, and structural word order errors. They emphasized the need for a flexible evaluation approach, recognizing multiple correct solutions beyond gold-standard sentences. Xu et al. [33] introduced the FCGEC corpus, which annotates grammatical errors such as Incorrect Word Collocation, Component Missing, Component Redundancy, Structure Confusion, Incorrect Word Order, Illogical, and Ambiguity, from operational and semantic perspectives. This fine-grained categorization enhances the understanding of common Chinese errors. Wang et al. [13] analyzed 66 sentences, identifying 100 errors across eight categories from the HSK learner corpus: character-level spelling errors, word selection errors, redundant word errors, missing word errors, and various sentence-level errors. Their evaluation of correction results from pre-trained models showed that BART was the most effective, particularly in handling character-level and miscellaneous sentence-level errors. T5 performed better than BERT on missing word and word order errors, likely due to its pre-trained decoder.

Despite the progress made in these studies, there are still several notable shortcomings. First, the human-annotated datasets are relatively small, which limits the robustness and generalizability of the findings. Second, although the error types are described as fine-grained, they are still quite dealing with the common operational categories used in general CGEC research—missing, replacing, redundancy, and word order. This categorization might not fully address the unique challenges posed by learning Chinese as a foreign language. A more nuanced corpus linguistics approach, specifically tailored to the intricacies of Chinese grammar and usage, could provide deeper insights and lead to more effective improvements in CGEC model performance.

## Methods

### Datasets

This study utilizes three publicly available CGEC evaluation datasets: NLPCC18 (https://github.com/zhaoyyoo/NLPCC2018_GEC), CGED-2021 (https://github.com/blcuicall/cged_datasets), and YACLC-Minimal (Track 3 dev) (https://github.com/blcuicall/CCL2022-CLTC). These datasets were contributed by the NLPCC-2018, the series of CGED shared tasks, and CCL2022- CLTC, and are used respectively to evaluate the performance of large language models. NLPCC18 test data is extracted from PKU Chinese Learner Corpus, a collection of essays written by foreign college students. It was annotated by two annotators with the first annotator marking the edit alone, and the second annotator checking the annotation and making a revision. This study takes the second annotator’s reference as the gold data to evaluate the LLMs’ performance. CGED-2021 is one of the series of shared CGEC task datasets, from 2020 and onwards mainly from Beijing Advanced Innovation Center for Language Resources at Beijing Language and Culture University. CCL2022-CLTC refers to the Chinese Learner Text Correction (CLTC) task in Chinese Computational Linguistics conference. This study takes the development dataset of track 3 in this task called YACLC-Minimal. Sentences in datasets of NLPCC18 and CGED have only one reference but CCL2022-CLTC offers multiple corrections per sentence, thereby providing a more comprehensive basis for evaluating CGEC systems. The annotation guidelines follow the general principle of Minimum Edit Distance. Errors are divided into four types: redundant words (denoted as a capital “R”), missing words (“M”), word selection errors (“S”), and word ordering errors (“W”). All the datasets used in the experiment are described in Table 1.

**Table 1:** Test datasets statistics.

Datasets	No. of Sentence	No. of Reference
NLPCC18	2000	1983(99.15%)
CGED-2021	2296	2296(100%)
YACLC-Minimal (Track 3 dev)	1839	15,935(99.98%)

### Evaluation

To evaluate the capabilities of LLMs for CGEC, we prompt three top LLMs of varying sizes, including GPT-4 (GPT-4-0613, up to Sep 2021), LLaMA-3-70B, and ERNIE4.0. For experiments with ChatGPT, we use the official API to prompt GPT-4. For those with LLaMA-3-70B and ERNIE4.0, we use the Qianfan LLM Platform, which is provided by Baidu in China. The prompt is direct and to the point: “请使用中文回答问题。请根据最小编辑原则修改以下句子中的语法错误,并直接仅给出修改后的正确语句, 不需要任何解释:/Please answer the questions in Chinese. According to the principle of minimal edits, please correct the grammatical errors in the following sentence and provide only the corrected sentence directly, without any explanation:”.

For assessment purposes, we employ the MaxMatch (M2) metric, as developed by Dahlmeier and Ng [34], which leverages Levenshtein distance to align the original and corrected sentences and identifies the most extensive matching edits to calculate precision, recall, and the F score. To calculate the metrics in Chinese, an adaptation of the F1 score that emphasizes precision by weighting it twice as heavily as recall, underscoring the prevailing view in grammatical error correction research that precision is more crucial than recall. To guarantee uniformity in our evaluations, we utilize the available MaxMatch scorer (https://github.com/nusnlp/m2scorer) for CGEC.

### Corpus linguistics analysis

In the study, we conducted a one-way Analysis of Variance (ANOVA) to compare the differences in editing distance values among ChatGPT, Llama-3, Ernie4 (Wenxinyiyan from Baidu), and human annotators. The ANOVA was performed using R (version 4.3.2) with the aov function, ensuring that our statistical analysis is both transparent and reproducible. Prior to the ANOVA, we verified the assumptions of normality and homogeneity of variances using the Shapiro-Wilk test and Levene’s test, respectively. Post-hoc comparisons were conducted using Tukey’s Honest Significant Difference (HSD) test to identify specific group differences where significant effects were found. Detailed scripts and data used for these analyses are provided in the supplementary materials, further enhancing the reproducibility of our findings. This comprehensive statistical approach allows for a robust comparison of the editing behaviors of different LLMs against human performance, thereby reinforcing the validity of our results.

Moreover, we conducted a detailed comparative keyword and key n-gram analysis using Quanteda package in R to evaluate the performance of the LLMs from a linguistic perspective. Since the differences between incorrect and corrected sentences in the datasets are primarily grammatical—while content words largely remain unchanged—this analysis effectively focuses on grammatical word usage. We processed the corrected outputs from the LLMs, the corrections made by human annotators, and the original learner sentences to extract word and n-gram frequencies. Using chi-squared (χ^2^) tests within R, we determined the significance of differences in the frequency of key linguistic devices among learners, annotators, and LLMs, thereby identifying specific grammatical features that distinguish the groups. The combination of corpus analysis with statistical testing provided a robust methodological framework for evaluating the linguistic performance of LLMs. All scripts, frequency lists, and statistical outputs have been documented and are available in the supplementary materials to enhance the transparency and reproducibility of our findings.

## Results

### LLMs performance

Table 2 presents the performance of three LLMs–GPT-4, ERNIE-4.0-8k, and LLAMA-3 70B –across different datasets for CGEC. In the YACLC-Minimal dataset with multiple annotators, ERNIE-4.0-8k exhibits notably higher performance. Meanwhile, it achieves the highest precision, recall, and F0.5 in CGED-2021 dataset with a single annotator. ERNIE-4’s robust performance underscores its suitability for precise grammatical error correction tasks in contexts of Chinese as a foreign language. In contrast, GPT-4 and LLAMA-3 70B LLMs demonstrate lower scores, highlighting potential challenges in CGEC. ERNIE-4’s stronger performance may be due to its development in China and training on extensive Chinese language data.

**Table 2:** Performance comparison among LLMs with minimum editing.

Test Datasets	LLMs	ExactMatch (character level)		
		P	R	F0.5
YACLC-Minimal (Track 3 dev) (multiple annotators)	GPT-4	41.75	50.37	43.23
	ERNIE-4.0-8k	**55.69**	**52.88**	**55.10**
	LLAMA-3 70B	41.55	44.59	42.13
YACLC-Minimal (Track 3 dev) (annotator T0)	GPT-4	18.51	22.29	19.16
	ERNIE-4.0-8k	**25.34**	**22.63**	**24.75**
	LLAMA-3 70B	17.55	17.77	17.59
CGED-2021	GPT-4	15.63	34.37	17.55
	ERNIE-4.0-8k	**28.85**	**37.72**	**30.28**
	LLAMA-3 70B	13.52	25.76	14.94
NLPCC18	GPT-4	16.79	27.16	18.18
	ERNIE-4.0-8k	**27.50**	**29.96**	**27.96**
	LLAMA-3 70B	15.27	21.20	16.17

In comparison to previous studies utilizing the character-based M2 metric, Zhang et al. [30] achieved an F0.5 score of 53.81 for NLPCC18 and 47.52 for the CGED test data. For the YACLC-Minimal (Track 3 dev), there are currently no comparative studies available; however, the reported F0.5 score on the YACLC-Minimal test data is also 47.52 [30], which can serve as a rough reference. The F0.5 scores for LLMs are significantly lower than those of state-of-the-art ensemble or complex seq2seq models. This discrepancy may be attributed to the large-scale mixed data used for training, predominantly in English, for models like GPT-4 and LLaMA-3 70B. While LLMs provide a more accessible solution for CGEC applications, their performance in terms of precision and recall is significantly lower and still falls short of human annotators.

### Corpus linguistic analysis

#### Editing distance

The main goal of the editing distance analysis is to show that LLMs have a tendency to make more corrections to sentences compared to human annotators. By comparing the Levenshtein distances, between the source texts and the predictions we can determine how editing each model does. We segment the corrected sentences into words instead of characters which allows us to calculate the minimum number of word changes needed to transform one string into another using the Levenshtein distance. This method gives us a way to assess the level of corrections made. After calculating the editing distance, we conducted a one-way ANOVA analysis to compare the difference of distance values among ChatGPT, Llama-3, Erinie4, and human annotators.

The ANOVA results on comparing the editing distances of wrong sentences and corrected sentences indicate significant differences among the various models and groups tested. The analysis shows that the Levenshtein distance is significantly higher for the GPT-4 model (mean difference = 2.13, 95% CI [1.86, 2.41], p < 0.0001) and the llama-3 model (mean difference = 1.81, 95% CI [1.54, 2.09], p < 0.0001) compared to the ERNIE-4 model. This suggests that GPT-4 and llama-3 make more substantial changes to correct sentences. Additionally, human corrections exhibit a significantly lower Levenshtein distance compared to GPT-4 (mean difference = -1.94, 95% CI [-2.22, -1.67], p < 0.0001), indicating that human corrections are more conservative and potentially more precise. Furthermore, there is a slight but significant difference between Llama-3 and GPT-4 (mean difference = -0.32, 95% CI [-0.60, -0.04], p = 0.016), highlighting nuanced variations in the correction approaches of these models. Overall, these results indicate significant differences in the editing distances between most pairs of groups, except for the comparison between human and ERNIE-4 (mean difference = 0.19, 95% CI [-0.08, 0.47], p = 0.2807).

#### Keyword analysis

The keyword analysis using Quanteda in R revealed distinct patterns in the original texts produced by Chinese learners and the texts generated by the LLMs. By comparing these texts, we identified key differences that might provide valuable insights into CGEC research. The analysis highlighted several keywords that were significantly more frequent in the learners’ texts, human annotated texts, and LLMs’ texts respectively. These keywords may serve as indicators of common errors made by learners or LLMs, hence of the LLMs’ real performance.

Table 3 presents the top 20 keywords used in LLMs in contrast to the usage in learners’ original texts. It includes chi-squared values, p-values, and the frequency of keyword usage by learners and LLMs. By examining these keywords, we can categorize them based on their grammatical roles and semantic meanings, providing insight into how different groups utilize language elements in grammatical error correction. From a grammatical perspective, the keywords can be divided into several categories. Modals and particles, such as “会/will”, “将/will”, and “了/aspect marker”, indicating future actions, obligations, or completed actions, are essential for constructing grammatically correct sentences in Chinese. Conjunctions like “和/and”, “并/and”, “且/and”, and “如果/if” link clauses or phrases, facilitating complex sentence structures. Additionally, prepositions and locatives, exemplified by “中/in”, denote positions or locations within sentences. These grammatical roles highlight the structural differences in language use between learners and LLMs. Semantically, the keywords can be grouped based on their meanings and contextual use. Temporal and conditional words like “已经/already”, and “如果/if” provide information about time and conditions, situating actions within temporal and hypothetical contexts. Keywords such as “拥有/possess” and “需要/need” relate to the existence or necessity of objects or states, indicating ownership or requirement. Words like “却/but”, “不仅/not only”, and “仍然/still” express contrasts, additions, or continuity, adding complexity to statements. Keywords like “无法/unable” and “是否/whether” indicate abilities or possibilities, expressing capability or uncertainty. This semantic diversity suggests that LLMs engage with a wide array of contexts and scenarios, albeit with varying degrees of accuracy and sophistication. Chinese learners often use fewer modals, particles, and conjunctions, which can lead to mistakes in their writing. While LLMs aim to correct these deficiencies by incorporating more of these grammatical elements, their interventions can sometimes result in redundancy and overly complex expressions. This tendency highlights a gap between the concise writing style preferred by human learners and the more complex constructions produced by LLMs.

**Table 3:** Top 20 keywords featuring LLMs compared with learners.

keyword	chi2	p	learners	LLMs
会(will)	17.1313	0.0000	543	1974
如果(if)	14.1653	0.0001	176	718
和(and)	13.2579	0.0002	655	2290
无法(unable)	13.2049	0.0002	22	147
并(and)	12.358	0.0004	75	348
是否(whether)	10.6408	0.0011	13	98
中(in)	10.4039	0.0012	257	959
将(will)	10.3865	0.0012	42	214
已经(already)	10.2896	0.0013	113	470
都(all)	8.98991	0.0027	527	1819
都会(will)	8.30855	0.0039	22	126
看到(have seen)	8.11425	0.0043	73	314
需要(need)	7.39185	0.0065	73	309
仍然(still)	7.3104	0.0068	6	54
且(and)	7.2388	0.0071	5	49
拥有(possess)	6.8622	0.0088	7	57
非常(very)	6.5632	0.0104	145	548
了(particle)	6.4206	0.0112	1871	5954
却(but)	6.2834	0.0121	63	266
不仅(not only)	6.0368	0.0140	38	175

Tables 4 and 5 are the comparison between LLMs and human annotators, revealing distinct syntactic and semantic patterns in their usage of keywords. A notable difference is that human annotators tend to feature single Chinese characters, while LLMs more frequently use two-character Chinese words. This reflects the mini-editing strategy of human annotators, who make minimal edits to correct sentences. In contrast, LLMs may generate more substantial changes, using complete words to ensure grammatical correctness. Common keywords such as “和/and”, “并/and”, “无法/unable”, and “如果/if” are more frequently used by LLMs, indicating their tendency to create coherent sentences. LLMs might overuse the tense and aspect markers like “将/will”, “已经/already”, “正在/be+ving”, and “曾经/once” to clarify the timing of actions and ensure grammaticality even when unnecessary. LLMs tend to use more modals, particles, and conjunctions to produce fluent Chinese, which can result in redundancy and less concise expressions. In contrast, Chinese native speakers often prefer more straightforward constructions, prioritizing clarity and brevity. This difference in writing style suggests that while LLMs may generate grammatically correct sentences, they may lack the conciseness and precision that characterize human communication.

**Table 4:** Keywords featuring LLMs compared with human annotators.

keyword	chi2	p	annotators	LLMs
和/and	14.4525	0.00014	649	2290
无法/unable	14.2174	0.00016	21	147
并/and	13.5403	0.00023	73	348
如果/if	12.7146	0.00036	179	718
将/will	11.8525	0.00057	40	214
中/in	11.6103	0.00065	253	959
已经/already	9.72573	0.00181	114	470
是否/whether	8.42373	0.00370	15	98
非常/very	7.9299	0.00486	141	548
且/and	7.21797	0.00721	5	49
感到/feel	5.84564	0.01561	75	305
当/when	5.05798	0.02451	88	343
仍然/still	4.91560	0.02661	8	54
正在/be+ving	4.78629	0.02868	13	74
曾经/once	4.58063	0.03233	14	77

**Table 5:** Keywords featuring human annotators compared with LLMs.

keyword	chi2	p	annotators	LLMs
的话/if	22.3411	0.0000	214	431
跟/with	16.2660	0.0000	263	582
可/can	8.34640	0.0038	75	149
没/not	7.6317	0.0057	205	486
用/with	7.5401	0.0060	207	492
的/particle	7.5368	0.0060	8067	23247
亦/also	7.1539	0.0074	16	20
而且/and	6.7347	0.0094	193	461
要是/if	6.5698	0.0103	24	37
不只/not only	6.5300	0.0106	15	19
好多/many	6.4128	0.0113	19	27
有/have	6.3710	0.0116	1102	3008
很/very	5.7631	0.0163	861	2334
可是/but	5.6031	0.0179	140	329
要/want	5.4838	0.0191	412	1073
学/learn	4.6411	0.0312	190	471
并且(and)	4.3126	0.0378	40	80
使(make)	4.1439	0.0417	114	271
已(already)	3.9957	0.0456	52	111

Table 6 shows the keywords featuring human annotators in contrast to learners. Only 5 keywords are salient, which shows fewer variations and is the result of the human mini-editing strategy. Devices like the modal verb “会/will”, the particle “地/a particle used to form adverbial phrases”, and the inclusive adverb “都/all” show significant differences with annotators using it more frequently than learners.

**Table 6:** Keywords featuring human annotators compared with learners.

keyword	chi2	p	annotators	learners
会(will)	7.6742	0.0056	637	543
地(-ly)	6.4778	0.0109	354	290
遵守(obey)	4.7789	0.0288	13	4
都(all)	4.6141	0.0317	598	527
经历(experience)	3.9863	0.0458	45	28
了(aspect marker)	3.8582	0.0495	1989	1871

#### Key N-gram analysis

The key n-gram analysis depicted in Table 7 reveals a significant divergence in the usage patterns of key n-grams (n = 2, 3) between texts produced by learners and those generated by LLMs. Based on their grammatical functions and usage patterns, they could be grouped into several categories including frequency modifiers and intensifiers, expressions of possibility and probability, temporal context, actions and outcomes, descriptions and comparisons, and initiating actions. This grouping allows for a detailed examination of how these n-grams are used differently by LLMs and human learners.

**Table 7:** Key N-grams (n = 2,3) featuring LLMs compared with learners.

keyword	chi2	p	learners	LLMs
就会/will	13.0156	0.0003	27	167
变得/become	10.6151	0.0011	8	75
可能会/may	9.6837	0.0019	7	67
了很多/le +a lot	8.0390	0.0046	35	175
就不/then no	6.2227	0.0126	22	116
时候起/from + time	5.9429	0.0148	1	24
是我/is me	5.7377	0.0166	83	331
给了/gave	5.7112	0.0169	3	34
可以从/can from	5.1198	0.0237	0	19
不再/no longer	4.8573	0.0275	6	45
感受到/feel	4.8573	0.0275	6	45
会觉得/will feel	4.8253	0.0280	2	26
了许多/le+many	4.7144	0.0299	5	40
就像/just like	4.5962	0.0320	6	44
会感到/will feel	4.4454	0.0350	5	39
每次都/every time	4.3154	0.0378	1	19
也会/also will	4.2695	0.0388	35	153

Table 7 shows that LLMs use certain n-grams much more frequently than learners. For instance, the n-grams for expressing changes and possibilities “就会/will”, “变得/become” and “可能会may” show higher usage by LLMs. N-grams indicating quantity or degree, such as “了很多 /le + a lot” and “了许多/le + many”, are also used more frequently. This indicates that LLMs are more adept at using phrases that convey intensity or extent. Additionally, n-grams like “是我 (is me)” and “时候起/from + time” show substantial differences, highlighting LLMs’ ability to generate self-referential constructs and temporal expressions more effectively. The frequent use of “给了/have given” and “会觉得/will feel” by LLMs further underscores their tendency to construct sentences with past actions and future feelings, contributing to richer narrative and descriptive language. This tendency suggests that LLMs excel in generating language that conveys complexity, which may not be as prevalent in the writing of Chinese L2 learners. However, this increased usage of certain n-grams can also lead to expressions that feel overly formal, potentially detracting from clarity.

Tables 8 and 9 list the key n-grams featuring LLMs and human annotators, respectively. It is apparent that LLMs have much fewer key n-grams, whereas human annotators have more featured phrases. For LLMs, these phrases indicate that LLMs tend to use specific grammatical structures to express changes, conditions, time-specific actions, ongoing activities (“我正在/I am V+ing”), “当我/when I”, “就会/will”, and “变得/become” in Table 8. In contrast, Table 9 reveals that human annotators frequently use n-grams that are more about detailed expressions and specific conditions. For example, “很多的/many”, “的所有的/all of”, and “有很多的/there are many” indicate their attention to specificity and possession. Additionally, annotators favor phrases like “起来是/seem to be”, reflecting their focus on clear and descriptive language. This disparity highlights that while LLMs tend to favor more formulaic constructions, human annotators are more adept at crafting nuanced and contextually rich expressions. The variety in human-generated n-grams suggests a greater awareness of subtleties in meaning and context, which may contribute to clearer communication.

**Table 8:** Key N-grams (n = 2,3) featuring LLMs compared with human annotators.

keyword	chi2	p	annotators	LLMs
了许多/have V+ed + a lot	6.0093	0.0142	4	40
我正在/I am V+ing	4.5028	0.0338	2	25
当我/when I	4.2552	0.0391	17	87
就会/will	4.1922	0.0406	39	167
变得/become	4.1706	0.0411	14	75

**Table 9:** Key N-grams (n = 2,3) featuring human annotators compared with LLMs.

keyword	chi2	p	annotators	LLMs
很多的/many	17.1073	0.0000	26	26
的所有的/all of	8.4530	0.0036	7	3
有这些/have these	8.2733	0.0040	4	0
起来是/seem to be	8.2733	0.0040	4	0
也没有了/also no more	5.4037	0.0201	3	0
到我死/until I die	5.4037	0.0201	3	0
常常在/often in	5.4037	0.0201	3	0
很多了/much more	5.4037	0.0201	3	0
我下决心/I decide	5.4037	0.0201	3	0
的产生/the generation	5.4037	0.0201	3	0
非常地/extremely	5.4037	0.0201	3	0
下决心/decide to	5.3528	0.0207	4	1
而没有/but no	5.3528	0.0207	4	1
跟我/with me	4.3001	0.0381	40	80
有很多的/there are many	4.2004	0.0404	9	11

Table 10 offers a comparative analysis of key n-grams used by human annotators versus learners. Notably, only two phrases are featured for human annotators, suggesting that they employ minimal edit strategies and concentrate on correcting word errors rather than phrases. This approach contrasts sharply with the editing strategies of LLMs, which tend to generate more complex phrases, highlighting a fundamental difference in how each group addresses grammatical corrections.

**Table 10:** Key N-grams (n = 2,3) featuring human annotators compared with learners.

keyword	chi2	p	learners	LLMs
可以从/can… from	4.1756	0.0410	6	0
每次都/every time	4.0107	0.0452	8	1

## Discussion

This study shows that the CGEC performance of LLMs, measured by the MaxMatch metric, is significantly lower than that of current state-of-the-art models, such as ensemble and sequence-to-edit approaches, which contradicts findings from previous studies [19]. From a corpus linguistic perspective, the underlying reasons for this inferior performance are primarily linguistic in nature. Based on the editing distance analysis results, LLMs have significant differences in the editing distances from human annotators. In addition, the keyword and key n-gram analysis findings from the comparison between LLMs and human annotators provided apparent evidence that LLMs might have significant over-correction in dealing with words instead of phrases and over-complication with sentence structures. Using these words and phrases as probes, LLMs’ performance in syntactic and semantic dimensions was qualitatively analyzed in this section.

Seq2Seq models tend to generate sentences with higher probabilities and replace infrequent words with more frequent ones, leading to overcorrection [9]. Alirector in Yuan and Quan’s study [9] is an example to mitigate the number of overcorrections without deteriorating under-correction by distilling knowledge and finetuning LLMs. However, their analysis focuses on four common operational types of errors. In this study, we shifted focus from operational errors to specific linguistic errors and found out that conjunctions, particles, and modal verbs are more frequently used by LLMs than human annotators. Both LLMs and human annotators employ those keywords to establish logical relationships, convey temporal and aspectual information, and indicate degree and intensity. However, LLMs show a strong tendency to overuse them to generate more coherent and complex sentence constructions, as shown in Table 11, which can be beneficial in formal contexts where detailed explanations are required. It can also lead to potential overcorrection, which may not always align with the natural flow of conversational or written Chinese as produced by learners. The results align with the word-level analysis from previous studies [13, 23], providing clearer insights into the nature of LLMs. LLMs tend to generate more formal and structured text, likely reflecting the influence of their training data, which often consists of more formal texts. In contrast, human annotators prioritize simplicity and clarity, which are essential for effective communication. Additionally, this tendency for LLMs to overuse certain words does not extend to phrases, as the analysis of key n-grams reveals that LLMs do not utilize more complex n-grams for error correction compared to annotators. Several featuring n-grams are similar with keywords showing temporal actions.

**Table 11:** LLMs’ Overuse of linguistic devices.

Source	他们只是想要解渴, 没有想将来会发生什么事情。
	They just wanted to quench their thirst, without thinking about what might happen in the future.
ERNIE-4	他们只是想要解渴, 没有想将来会发生什么事情。
	They just wanted to quench their thirst, without thinking about what might happen in the future.
ChatGPT-4	他们只是想解渴, **并**没有想**到未来**会发生什么事情。
	They just wanted to quench their thirst and did not consider what might happen in the future.
Llama-3	他们只是想要解渴, 没有想**到**将来会发生什么事情。
	They just wanted to quench their thirst, without considering what might happen in the future.
Human	他们只是想要解渴, 没有想将来会发生什么事情。
	They just wanted to quench their thirst, without thinking about what might happen in the future.
Source	老**家从来**给我的安全感会完全**泯灭**。
	The sense of security that my hometown used to provide me will completely fade away.
ERNIE-4	老家给我的安全感**从来**会完全泯灭。
	The sense of security that my hometown has always given me will completely fade away.
GPT-4	**我的**老家给**予**我的安全感**从来不会**完全泯灭。
	The sense of security my hometown has given me will never completely fade away.
Llama-3	老家**曾经**给我**带来的**安全感会完全泯灭。
	The sense of security that my hometown once brought me will completely fade away.
Human	老**房子一直以**来给我的安全感会完全**消失**。
	The sense of security that the old house has always given me will completely disappear.

In terms of under-correction, LLMs struggle with semantic understanding and usage of modal verbs and collocations. Albeit the overuse of modal verbs, LLMs fail to understand fully the complex and subtle meaning of different modal verbs. For example, the negative modal verbs of “不可以/not to be allowed to”, “不能/unable to”, and “无法/cannot” in Table 12 are misused by both learners and LLMs. The reason is that the word “会/know how to” has a similar meaning to “能/to be able to” and “可以/to be allowed to”, which are all often translated as “can” in English. This is a useful way of thinking about them, but their usage does overlap because of the context. In this context, their negative forms involve subtleties in meaning with co-occurrence of the verb “融化/melt” that are difficult for LLMs to accurately interpret and correct. For collocational knowledge, researchers hold that word embedding technology provides a great possibility for LLMs to learn natural word occurrence. However, in the CGEC task, original sentences are written by L2 learners, more than 1 or 2 errors in the sentence, and cause LLMs to fail to understand the original meaning of the writer then fail to perform well in collocational error correction. For example, the error in Table 12 is the mis-collocation of “组成/constitute” and “人生的小部分/a small part of life”. The central word of the noun phrase is “小部分/ a small part” in syntax, and “组成/constitute” seems to collocate well with it. However, the real collocate of the verb in this sentence should be “人生/life” and “完成/accomplish” is proper in the verb position to form a natural and idiosyncratic unit of meaning. Another typical collocational error is the missing noun object of the verb “策划/plan”, and none of the LLMs can grasp the meaning to add the object “活动/event” though they make other over-corrections. This aligns with previous studies [33] and it is clear that the performance on word collocation is inferior and the performance of LLMs among various fine-grained grammatical errors will be very challenging [31].

**Table 12:** LLMs’ typical under-correction instances.

Source	因为天气冷, 公路上的雪**不可以**融化。
	Because of the cold weather, the snow on the road is not allowed to melt.
ERNIE-4	因为天气冷, 公路上的雪**无法**融化。
	Because of the cold weather, the snow on the road is unable to melt.
ChatGPT-4	因为天气冷, 公路上的雪**无法**融化。
	Because of the cold weather, the snow on the road is unable to melt.
Llama-3	因为天气冷, 公路上的雪**不能**融化。
	Because of the cold weather, the snow on the road cannot melt.
Human	因为天气冷, 公路上的雪**不会**融化。
	Because of the cold weather, the snow on the road will not melt.
Source	没有勇气的人生, 就连人生的小部分也不能**组成**。
	Without courage, even a small part of life cannot be constituted.
ERNIE-4	没有勇气, 就连人生的小部分也无法组成。
	Without courage, even a small part of life cannot be constituted.
ChatGPT-4	没有勇气的人生, 连人生的一小部分也不能组成。
	Without courage, even a small part of life cannot be constituted.
Llama-3	没有勇气的人生, 就连人生的一小部分也不能组成。
	Without courage, even a small part of life cannot be constituted.
	Without courage, even a small part of life cannot be constituted.
Human	没有勇气的人生, 就连人生的小部分也不能**完成**。
	Without courage, one cannot even accomplish a small part of life.
Source	**策划了**包琳和赵颖非常辛苦了!
	Planning Bao Lin and Zhao Ying worked very hard!
ERNIE-4	策划了包琳和赵颖**他们**非常辛苦!
	Planning Bao Lin and Zhao Ying, they worked very hard!
ChatGPT-4	**策划**包琳和赵颖, **大家**非常辛苦了!
	Planning Bao Lin and Zhao Ying, everyone worked very hard!
Llama-3	策划了包琳和赵颖非常辛苦!
	Planning Bao Lin and Zhao Ying worked very hard!
Human	策划了**这次活动**的包琳和赵颖辛苦了!
	Bao Lin and Zhao Ying, who planned this event, worked very hard!

For hallucination, we focus on semantic inconsistency and common-sense errors. Prior studies evaluating LLMs’ hallucination have shown that LLMs provide incorrect or misleading information [35, 36]. In CGEC, semantic inconsistency is one major type of incorrect and misleading information, which occurs when the corrected sentence adds unrelated content or changes the original meaning in a way that diverges from the source sentence. For instance, as shown in Table 13, the source sentence has a redundant “曾经刚毕业/once just graduated,” which ERNIE-4, ChatGPT-4, and Llama-3 all simplify to “曾经毕业/once graduated,” thus removing redundancy but altering the intended meaning. In contrast, the human correction retains “刚毕业/just graduated,” clearly indicating a recent event. Additionally, another hallucination is a common-sense error happening when the generated content does not align with real-world knowledge. For example, LLAMA-3’s corrected sentence contains the awkward phrase “骑着滑板/riding a skateboard,” which is a miscollocation that defies common sense. ChatGPT-4 avoids the common sense error but introduces a new subject “有人/someone,” thereby changing the original meaning of the source sentence and making the semantic consistency hallucination. Our results support the work of other studies [37]. A key reason for this issue seems to be that the source texts consist of isolated sentences, lacking the necessary context from preceding and following sentences. This absence of contextual information restricts LLMs’ ability to accurately interpret and correct errors, often resulting in the generation of irrelevant or incorrect content. Chollampatt & Wang [38] showed that incorporating surrounding sentence context significantly improves GEC performance. To tackle the problem of hallucinations, researchers should focus on creating more training data with broader contexts, as well as corresponding test data. By integrating multi-sentence contexts in both training and evaluation phases, LLMs can better understand the relationships and dependencies between sentences, thereby enhancing their accuracy in making corrections.

**Table 13:** LLMs’ typical hallucination instances.

Source	有一天, 一个大学生玩着滑板, 在他眼前过去。
	One day, a college student was playing on a skateboard and passed in front of him.
ERNIE-4	有一天, 一个大学生玩着滑板, 从他眼前滑过。
	One day, a college student was playing on a skateboard and slid past him.
ChatGPT-4	有一天, 一个大学生**正在**玩滑板, **有人**从他眼前过去。
	One day, a college student was playing on a skateboard, and someone passed in front of him.
Llama-3	有一天, 一名大学生**骑着**滑板从他眼前过去。
	One day, a college student rode a skateboard past him.
Human	有一天, 一个大学生玩着滑板, 从他眼前经过。
	One day, a college student was playing on a skateboard and passed by him.
Source	他今年25岁, **曾经刚**毕业于泰国曼谷商业大学。
	He is 25 years old this year and once just graduated from Bangkok University of Commerce in Thailand.
ERNIE-4	他今年25岁, **曾经**毕业于泰国曼谷商业大学。
	He is 25 years old this year and once graduated from Bangkok University of Commerce in Thailand.
ChatGPT-4	他今年25岁, **曾经**毕业于泰国曼谷商业大学。
	He is 25 years old this year and once graduated from Bangkok University of Commerce in Thailand.
Llama-3	他今年25岁, **曾经**毕业于泰国曼谷商业大学。
	He is 25 years old this year and once graduated from Bangkok University of Commerce in Thailand.
Human	他今年25岁, **刚**毕业于泰国曼谷商业大学。
	He is 25 years old this year and just graduated from Bangkok University of Commerce in Thailand.

## Conclusion

The corpus linguistic analysis of LLMs’ performance in CGEC has provided valuable results and insights into their strengths and limitations. The distance editing analysis, keyword analysis, and key n-gram analysis demonstrated that LLMs tend to overcorrect the source sentences, often at the word level rather than the phrase level errors. Further manual analysis identified LLMs’ instances of over-corrections, under-corrections, and hallucinations, highlighting specific areas where LLMs diverge from human annotators.

These findings have a clear vision for the development of using LLMs in CGEC. To enhance the performance, it might be essential to fine-tune these models with more diverse and contextually rich training data that emphasize collocational, modal, and semantic corrections. Additionally, implementing feedback loops where the model learns from human annotators’ corrections can help LLMs develop a deeper understanding of context and improve their overall accuracy. These approaches will likely lead to more natural and effective language corrections, aligning LLM output more closely with human annotators.

The results indicate several practical strategies for enhancing LLMs’ performance in CGEC tasks. First, fine-tuning these models with diverse and contextually rich training data that emphasizes collocational, modal, and semantic corrections is essential. Additionally, integrating collocational data and knowledge graphs can significantly improve their accuracy and reduce hallucinations. Collocational data provides LLMs with a better grasp of contextually appropriate word combinations, minimizing semantic errors. Meanwhile, knowledge graphs, which illustrate the relationships between entities, can help refrain the models’ responses from generating unrelated content. These enhancements could lead to more reliable and context-aware grammatical error corrections.

For the limitation of the study, the qualitative analysis is limited to a small sample of LLMs’ generated texts, and the analysis of LLMs’ linguistic performance is not comprehensive. This constrained scope means that the findings may not fully capture the breadth of potential errors and hallucinations that LLMs can produce. Additionally, the study primarily focuses on specific types of grammatical errors, potentially overlooking other significant aspects of linguistic performance such as style and pragmatic appropriateness. Future research should aim to include a larger and more diverse dataset of generated texts and consider a wider range of linguistic features to provide a more holistic evaluation of LLMs’ capabilities and limitations.

## Supporting information

S1 Data This zip file contains the R code along with all the related data and tools utilized in this study. (RAR)
